# Association of Serum Total Bilirubin Level With Abdominal Aortic Calcification: A Population-Based Cross-Sectional Study

**DOI:** 10.1155/mi/5229580

**Published:** 2025-07-27

**Authors:** Chunjiang Liu, Kuan Li, Guohua Wang, Ziqian He, Suyan Cao

**Affiliations:** ^1^Department of General Surgery, Shaoxing People's Hospital (The First Affiliated Hospital, Shaoxing University), No. 568 Zhong Xing Road, Shaoxing, Zhejiang, China; ^2^Department of Hepatobiliary Surgery, Kunshan Hospital of Traditional Chinese Medicine, No. 388 Zuchongzhi Road, Kunshan, Jiangsu, China; ^3^Department of Cardiology, Wuxi No. 2 People's Hospital (Jiangnan University Medical Center), No. 68 Zhongshan Road, Liangxi District, Wuxi, Jiangsu 214000, China

**Keywords:** abdominal aortic calcification, American, cross-sectional study, severe abdominal aortic calcification, total bilirubin level

## Abstract

**Objective:** The purpose of our study was to examine the association between serum total bilirubin level and abdominal aortic calcification (AAC) in the general United States population.

**Methods:** We analyzed data from the 2013–2014 National Health and Nutrition Examination Survey (NHANES) to assess the association of total bilirubin levels with AAC and severe AAC (SAAC). Restricted cubic spline (RCS) plots, weighted multivariable logistic regression (odds ratios [ORs] and 95% confidence intervals [CIs]), and stratified subgroup analyses (by age, sex, hypertension, diabetes mellitus, and body mass index [BMI]) were conducted.

**Results:** Our analysis included a total of 3016 participants. First, the RCS plots showed the U-shaped curve association of serum total bilirubin level with prevalence of AAC and SAAC. RCS analysis revealed a U-shaped association between serum total bilirubin levels and the prevalence of both AAC and SAAC. Serum total bilirubin levels were categorized into quartiles: Q1 (0.10–0.50 mg/dL), Q2 (0.51–0.60 mg/dL), Q3 (0.61–0.80 mg/dL), and Q4 (0.81–2.20 mg/dL). Second, after adjusting for potential confounders, compared with the Q1 group, the ORs with 95% CI for the association of total bilirubin level with AAC and SAAC across Q2, Q3, and Q4 were (0.71 (0.61, 0.98), 1.11 (0.90, 1.38), and 1.36 (1.07, 1.73) and 0.78 (0.58, 1.21), 1.12 (0.77, 1.65), and 1.28 (0.87, 1.77)), respectively. Finally, this U-shaped correlation was found among participants in age ≥60 years, with hypertension, with DM and with BMI of ≥30 kg/m^2^.

**Conclusions:** Our study identified a significant U-shaped association between serum total bilirubin levels and both AAC and SAAC. These findings suggested that monitoring and optimizing total bilirubin levels may offer potential preventive benefits against AAC development.

## 1. Introduction

Vascular calcification (VC), characterized by the deposition of calcium minerals in arterial walls, results from dysregulated mineral metabolism, chronic inflammation, and oxidative stress [1]. This pathological process predominantly manifests as coronary artery calcification and abdominal aortic calcification (AAC) [2]. Notably, AAC serves as a reliable predictor of cardiovascular events and an effective marker of subclinical atherosclerosis [3–6]. Moreover, severe AAC (SAAC) is independently associated with increased cardiovascular morbidity and mortality [7].

Oxidative stress and inflammation exhibit a bidirectional relationship, wherein each process may exacerbate the other [8]. This oxidative stress-induced inflammatory response has been implicated as a potential mechanism underlying VC [9]. Previous studies have identified significant associations between specific inflammatory and oxidative stress markers, including gammaglutamyl transferase (GGT) and high-sensitivity C-reactive protein (hs-CRP), and various metabolic conditions. These markers demonstrate strong correlations with obesity, type 2 diabetes, cardiovascular disease, and all-cause mortality [10–15]. Notably, these chronic conditions are themselves closely linked to the development of VC. The metabolic pathway of bilirubin production is intrinsically linked to oxidative stress regulation [16]. Bilirubin biosynthesis occurs via heme degradation, sequentially catalyzed by heme oxygenase and biliverdin reductase. As the terminal product of heme catabolism, serum total bilirubin levels demonstrate significant alterations in various metabolic disorders, including oxidative damage in atherosclerotic plaques and diabetic retinopathy [17]. Functioning as a potent endogenous antioxidant, bilirubin effectively scavenges oxygen free radicals generated during metabolic processes and inhibits low-density lipoprotein oxidation. These mechanisms contribute to atheroprotection and may modulate the pathogenesis of coronary artery disease [18]. Despite these established relationships, evidence regarding the association between serum total bilirubin (as an oxidative stress marker) and AAC remains scarce. We postulate that this readily measurable hematological parameter may serve as a biomarker for systemic oxidative stress burden and demonstrate significant associations with both AAC and severe AAC (SAAC). The present study was therefore designed to investigate these potential associations, addressing this important knowledge gap in cardiovascular pathophysiology.

## 2. Material and Methods

### 2.1. Study Population

**Figure 1:**
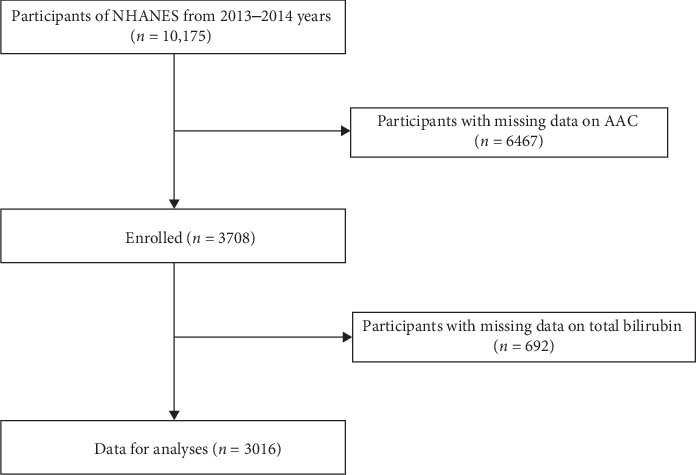
Study flowchart. AAC, abdominal aortic calcification; NHANES, National Health and Nutrition Examination Surveys.

The National Health and Nutrition Examination Survey (NHANES) database is an ongoing U.S. national population-based nutrition and health survey. It uses complex, multi-stage, and probability sampling techniques rather than a simple random sample based on the U.S. population. The survey covers all geographical regions of the United States to ensure that the sample is nationally representative [19]. We removed 6467 participants with missing AAC data, and 692 people with missing measures of serum total bilirubin data from the 3708 eligible individuals. Finally, this study included a total of 3016 individuals aged 20 years and older (Figure 1). The protocol of NHANES was approved by the National Center for Health Statistics (NCHS) research ethics review board, and that all participants signed informed consent forms [20]. More information about the data can be found at (https://www.cdc.gov/nchs/nhanes/index.htm).

### 2.2. Covariates

We followed the methods of [21, 22]. All the following covariate in the present study include: Demographic data, test results, survey results, dietary data, and lab results. Age, sex, family poverty income ratio (PIR), race/ethnicity, marital status, and education level was all included in the demographic data. Body mass index (BMI), and waist circumference were examined data. Questionnaire results included information on smoking status, drinking status, the complication of heart attack, congestive heart failure (CHF), angina pectoris, coronary heart disease (CHD), and stroke. Dietary data included mean energy intake, calcium intake, and phosphorus intake. Finally, white blood cells (WBC), neutrophil (Neu), lymphocyte (Lym), monocyte, red cell distribution width (RDW), mean cell volume (MCV), platelet, mean platelet volume, glycosylated hemoglobin (HbA1c), fast glucose (FBG), triglyceride (TG), total cholesterol (TC), high-density lipoprotein-cholesterol (HDL-C), serum uric acid (sUA), blood urea nitrogen (BUN), estimated glomerular filtration rate (eGFR), serum creatinine (Scr), total bilirubin, alkaline phosphatase (ALP), albumin (Alb), GGT, serum iron, serum calcium, and phosphorus were measured in the laboratory.

### 2.3. Measurement of Hematological Indicators

Serum specimens are processed, stored (−30°C), and shipped to the National Center for Environmental Health for testing. An in-depth description of how to collect and process instructions are provided in the NHANES Laboratory/Medical Technologists Procedures Manual. Professional technicians operated and used the automated hematology analyzing devices (Colter DxH 800 analyzer) to measure the results of the blood count (WBC, Neu, Lym, monocyte, MCV, RDW, platelet counts, and mean platelet volume), and the UniCel DxC 800 Synchron Clinical System (Beckman Colter, Brea, California) and the Beckman Colter UniCel DxC 660i Synchron Access chemistry analyzers to measure the results of total bilirubin, ALP, Alb, and GGT. Finally, in this study, we calculated neutrophil-to-lymphocyte ratio (NLR), platelet-to-lymphocyte ratio (PLR), neutrophil-to-albumin ratio (NAR), systemic immune inflammation (SII) index, and system inflammation response index (SIRI) for each participant as follows [23]: NLR = Neu count ( ×10^9^/L)/lLym count ( ×10^9^/L); PLR = platelet count ( ×10^9^/L) /Lym count ( ×10^9^/L); NAR = Neu count ( ×10^9^/L)/Alb (g/L); SII index ( ×10^9^/L) = Neu count ( ×10^9^/L)/Lym count ( ×10^9^/L) × platelet count ( ×10^9^/L); SIRI ( ×10^9^/L) = Neu count ( ×10^9^/L) × monocyte ( ×10^9^/L)/Lym count ( ×10^9^/L).

### 2.4. The AAC Measurement

AAC obtained by scanning the lumbar spine (including vertebrae L1–L4) using dual-energy X-ray absorptiometry (DXA, Densitometer Discovery A, Hologic, Marlborough, MA, USA) and then quantifying it using the Kauppila scoring system, varied from 0 to 24, with higher scores indicating greater calcification [24, 25]. The Kauppila scoring method evaluates calcification severity in various segments of the aortic walls corresponding to the region anterior to the lumbar vertebral L1–L4 [26]. During data collection at NHANES 2013–2014, DXA scans could not be performed in individuals who were under 40 years of age, pregnant, had used contrast (barium) within the past 7 days, weighed more than 450 pounds, and had scoliosis. An AAC score equal to 0 was no calcification, an AAC score greater than 0 and less than or equal to 6 was mild-moderate calcification, and an AAC score greater than 6 was severe calcification [27–29].

### 2.5. Statistical Analysis

In the study, all statistical analyses were performed using R version 4.4.0 (R Foundation for Statistical Computing, Vienna, Austria), and SPSS version 22.0 (SPSS Inc., Chicago, IL, USA). There was statistical significance at the *p*-value <0.05. The mean ± standard deviation was used to express continuous variables, and categorical variables were expressed as frequencies and percentages. The weighted Student's *t*-test and weighted chi-square test were performed to compare the continuous variables, and constituent ratios between each group, respectively. The restricted cubic spline (RCS) plot, and multivariate logistic regression analysis were performed to explore the potential nonlinearity of the association between serum total bilirubin level and prevalence of AAC and SAAC. In the study, to detect multicollinearity, the variance inflation factor (VlF) analysis is applied to evaluate all the candidate variables [30]. VIF values are variance inflation factors that measure the severity of multicollinearity. It is generally believed that the VIF value is greater than 5, and there is a multicollinearity problem [31]. A total of 3 models were constructed for adjusted (Model 1, Model 2, and Model 3). First, Model 1 was adjusted for age and sex. Second, Model 2 was further adjusted for family PIR, education level, race/ethnicity, marital status, the complication of hypertension, and DM, smoking status, and drinking status. Finally, Model 3 was further adjusted for BMI, waist circumference, the complication of heart attack, CHF, angina pectoris, CHD, stroke, hyperlipemia, and CKD, Alb, ALP, mean energy intake, calcium, and phosphorus intake, HbA1c, Scr, sUA, FBG, serum calcium, and phosphorus, TC, eGFR, Lym, monocyte, RDW, MCV, platelet, mean platelet volume, serum iron, NLR, PLR, SII index, SIRI, TG, HDL-C, and BUN. Additionally, subgroup analysis stratified by age, sex, hypertension, DM, and BMI was applied to examine the link between measures of serum total bilirubin level and prevalence of AAC and SAAC. Finally, in terms of sample size and statistical power, we also conducted a power analysis to determine the appropriate sample size needed to detect meaningful effects with sufficient power.

## 3. Results

### 3.1. Characteristics of Participants

The characteristics of the study population are shown in Table 1. This research comprised 1088 participants overall, who may be representative of the overall U.S. population of 117,719,728. Sex, family PIR, marital status, education level, smoker status, drink status, BMI, WBC, Neu, Lym, monocyte, MCV, RDW, platelet, PLR, Scr, NAR, SII index, Alb, GGT, serum iron, phosphorus, HDL, HbA1c, UA, and eGFR had significant difference among Q1–Q4 group.

### 3.2. Association of Serum Total Bilirubin Level With AAC and SAAC

**Figure 2:**
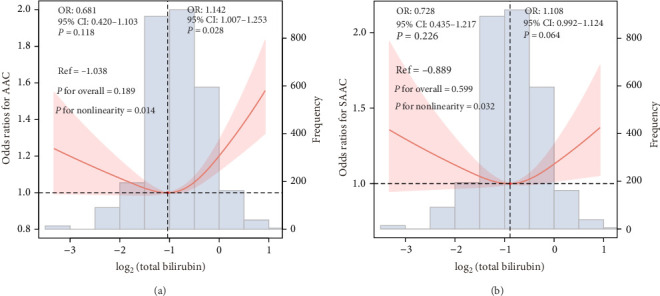
The restricted cubic spline plot of the association between serum total bilirubin level with the risk of (A) AAC and (B) SAAC. AAC, abdominal aortic calcification; SAAC, severe abdominal aortic calcification.

The collinearity analysis identified that high collinearity degrees for the VIFs of WBC, Neu, and NAR in Model 3 were all >5 (Supporting Information 1: Table S3 and Supporting Information 2: Table S4). The results of the multivariate logistic regression analysis can be found in Table 2. Serum total bilirubin levels were categorized into quartiles: Q1 (0.10–0.50 mg/dL), Q2 (0.51–0.60 mg/dL), Q3 (0.61–0.80 mg/dL), and Q4 (0.81–2.20 mg/dL). After adjusting for potential confounders, compared with the Q1 group, the odds ratios (ORs) with 95% CI for the association of total bilirubin level with AAC and SAAC across Q2, Q3, and Q4 were (0.71 (0.61, 0.98), 1.11 (0.90, 1.38), and 1.36 (1.07, 1.73) and 0.78 (0.58, 1.21), 1.12 (0.77, 1.65), and 1.28 (0.87, 1.77)), respectively. The RCS plot is shown in Figure 2A, representing a U-shaped curve correlation between serum total bilirubin level and the risk of AAC (*p* for nonlinearity = 0.014). Additionally, the RCS plot depicted in Figure 2B also illustrated a U-shaped correlation between serum total bilirubin levels and risk of SAAC (*p* for nonlinearity = 0.032).

### 3.3. Subgroup Analysis and Mediation Analysis

A subgroup analysis was conducted to further investigate the association of serum total bilirubin level with the prevalence of AAC and SAAC, stratified by age, sex, hypertension, and DM, and BMI (Supporting Information 3: Table S1 and Supporting Information 4: Table S2; Supporting Information 5: Figure S1 and Supporting Information 6: Figure S2). The U-shaped associations of serum total bilirubin level with AAC were found among participants in age <60 or ≥60 years, female, with hypertension, with DM and with BMI of <30 or ≥30 kg/m^2^. Additionally, the U-shaped association between serum total bilirubin levels and the risk of AAC were also observed among participants stratified by age (≥60 years), sex (male), with or without hypertension status, with DM status, and BMI (≥30 kg/m^2^). Additionally, we also explored whether lipid profiles (e.g., TC, LDL-C, HDL-C, and TG) mediate the association of total bilirubin with AAC and SAAC (Supporting Information 7: Figure S3 and Supporting Information 8: Figure S4). Only HDL-C showed a statistically significant indirect effect (*p*=0.048), suggesting it may partially mediate the relationship between total bilirubin and AAC, although the proportion mediated was relatively small (8.69%). There was no significant mediation effect observed for the other lipid parameters or in the association with SAAC.

## 4. Discussion

Studies have shown that inflammation and oxidative stress are the major drivers of VC [32–34]. Alterations in markers of inflammation and oxidative stress may influence the incidence and progression of AAC and SAAC. Therefore, this study examined the association between an oxidative stress marker (serum total bilirubin) and the prevalence of AAC and SAAC. Our results demonstrated a U-shaped relationship between serum total bilirubin levels and the risk of AAC and SAAC in the general U.S. population. According to Kang et al. [35] individuals with higher serum bilirubin levels exhibited a significantly lower incidence of coronary plaques and coronary artery stenosis, regardless of conventional cardiovascular risk factors. Zhang et al. [36] also established an independent inverse relationship between serum total bilirubin levels and coronary artery calcification scores in Korean men. Lower serum bilirubin concentrations could be considered a potential risk factor for coronary artery calcification in male populations [36]. Therefore, we hypothesized that bilirubin may suppress atherogenesis through inhibition of systemic inflammatory activity. Total bilirubin is an antioxidant under physiological conditions, inhibiting inflammation in the vasculature [37]. The U-shaped relationship between total bilirubin and VC may be attributed to the antioxidant and anti-inflammatory properties of bilirubin. Low levels of total bilirubin are associated with diminished antioxidant capacity and heightened inflammatory responses. Bilirubin possesses antioxidant properties, and reduced total bilirubin levels weaken its ability to neutralize oxidative stress. Consequently, the vascular wall is more susceptible to oxidative damage, leading to endothelial dysfunction and promoting the progression of VC. Additionally, total bilirubin exerts anti-inflammatory effects, and lower levels may exacerbate inflammatory responses. The release of various inflammatory factors by activated inflammatory cells can induce phenotypic transformation of vascular smooth muscle cells and osteoblasts, thereby, facilitating VC [38]. Additionally, total bilirubin is closely related to lipid metabolism and can affect the dissolution and excretion of cholesterol. Moderate bilirubin levels may help maintain the stability of blood lipid levels, reduce lipid deposition on the vessel walls, and thereby, lower the risk of atherosclerosis and calcification. However, when the bilirubin level is too low, this protective effect may weaken [36]. Finally, total bilirubin may regulate VC by influencing the phenotypic transformation and calcification process of vascular smooth muscle cells. In vitro experiments have shown that bilirubin can inhibit the calcification of vascular smooth muscle cells, which may be one of the mechanisms by which it has a protective effect on AAC [39]. In line with our findings, Nilsen et al. [40] reported that elderly patients with a recent myocardial infarction who had low bilirubin concentrations (<9 µmol/L) experienced a higher incidence of nonfatal cardiovascular events or death. Gullu et al. [41] demonstrated that higher serum bilirubin levels are associated with protection against coronary flow reserve impairment, coronary microvascular dysfunction, and potentially coronary atherosclerosis. Moreover, Akboga et al. [42] demonstrated that serum total bilirubin levels were independently and inversely related to the severity of coronary atherosclerosis in patients with stable CAD. Additionally, total bilirubin levels exhibited an inverse correlation with CRP, NLR, and RDW. These findings indicate that, in addition to its known impact on oxidative stress, higher serum total bilirubin levels may also exert an anti-inflammatory effect in the coronary atherosclerotic process [42]. In a sample of Chinese hypertensive patients, Wang et al. [43] observed a significant inverse correlation between serum total bilirubin and direct bilirubin levels and the risk of first ischemic stroke. Elevated levels of total bilirubin can induce abnormal liver function and exert toxic effects on bilirubin metabolism. High total bilirubin levels may serve as an indicator of impaired liver function. The liver plays a crucial role in lipid metabolism and vitamin D metabolism, among other processes. Dysfunction of the liver can disrupt these metabolic pathways, thereby, affecting the normal physiological functions of blood vessels and increasing the risk of VC. Additionally, excessive bilirubin may exert toxic effects on vascular endothelial cells, directly damaging them and leading to vascular endothelial dysfunction, which in turn promotes VC [38, 44, 45]. The research findings conducted by revealed Chen et al. [46] that elevated methylmalonic acid levels are linked to a greater occurrence of AAC. Similarly, a 2023 study by Wang and Zheng [47] reported a link between serum α-Klotho levels and SAAC, suggesting the involvement of endocrine factors in the calcification process. Serum α-Klotho may be a promising indicator for predicting the incidence and prognosis of cardiovascular disease. Meanwhile, they also found that within a sample of noninstitutionalized U.S. civilians, uric tobacco-specific Nitrosamine levels showed a positive correlation with the risk of SAAC when the concentration of uric NNN was below 1.354 ng/dL [48]. Additionally, Li et al. [49] found that a higher risk of AAC was closely associated with higher a body shape index (ABSI), and the discriminant ability of ABSI for AAC was significantly higher than that of height, weight, BMI, waist circumference, and waist-to-height ratio. In this study, we found that total bilirubin was independently associated with AAC and could serve as a supplementary biomarker for cardiovascular risk stratification. Although it is not ready to guide treatment yet, this potential can inspire future research, such as exploring whether increasing endogenous bilirubin or targeting related pathways may prevent VC.

The liver is the main site of drug metabolism, and many drugs are metabolized in the liver through enzyme systems such as cytochrome P450. When certain drugs that affect liver function are used, such as acetaminophen (which can cause liver cell damage in excess) and isoniazid, they may interfere with the normal metabolic function of the liver [50]. This metabolic disorder may lead to abnormal accumulation or clearance of certain metabolic products in the body, thereby, affecting the metabolic balance of minerals such as calcium and phosphorus and indirectly promoting the occurrence of VC [51]. Additionally, some drugs may interfere with the metabolism of vitamin D. The liver is an important site for the hydroxylation reaction of vitamin D, converting vitamin D into 25-hydroxyvitamin D, which is then converted into active vitamin D through the kidneys [52]. If the drug affects this function of the liver, it may lead to a reduction in the production of active vitamin D, thereby, affecting the absorption and utilization of calcium and increasing the risk of VC [53]. Some medications, such as diuretics (furosemide, etc.), may cause electrolyte imbalances, leading to abnormally elevated levels of blood calcium and phosphorus. The increase of blood calcium and blood phosphorus is one of the risk factors for VC. Excessive calcium and phosphorus are prone to deposit on the vessel walls, promoting the formation of VC [54]. The liver function of patients with chronic liver diseases is impaired, and they are unable to effectively convert vitamin D into its active form, resulting in a decrease in calcium absorption rate. Long-term insufficient calcium intake can lead to a decrease in blood calcium levels, which in turn maintains stable blood calcium levels by increasing the secretion of parathyroid hormone. However, this compensatory mechanism may cause excessive calcium loss in bones and increase the reabsorption of phosphorus by the kidneys, thereby raising blood phosphorus levels and promoting the occurrence of VC [55]. Chronic liver diseases are often accompanied by cholestasis or insufficient secretion, which affects the absorption of fats and fat-soluble vitamins (such as vitamin D and K). Vitamin K is an important factor regulating calcium metabolism. Insufficient absorption of it can affect the deposition and clearance of calcium on the vessel walls, increasing the risk of VC [56]. Patients with chronic liver diseases often have decreased levels of sex hormones, which not only accelerates bone mass loss but may also indirectly promote VC by affecting calcium metabolism and distribution [57]. Patients with chronic liver diseases have a long-term chronic inflammatory state in their bodies, with elevated levels of inflammatory factors. These inflammatory factors can induce vascular endothelial cells to express more calcium-binding proteins and calcium transporters, promoting the deposition of calcium ions on the vascular walls [58]. In patients with decompensated liver cirrhosis, hepatorenal syndrome is often concurrent, resulting in impaired renal function. The kidneys are important organs that regulate calcium and phosphorus metabolism. When renal function is impaired, the excretion of phosphorus decreases, blood phosphorus levels rise, and calcium reabsorption is also affected, resulting in a decrease in blood calcium. This disorder of calcium and phosphorus metabolism will further aggravate the occurrence of VC [59]. Chronic liver diseases lead to decreased liver function and a decline in the liver's ability to synthesize Alb. Alb has the functions of maintaining the osmotic pressure of plasma colloid and transporting various substances. Its reduction may result in a decrease in plasma osmotic pressure, extravasation of intravascular fluid, and at the same time affect the transport and distribution of minerals such as calcium and phosphorus, indirectly influencing VC. The NHANES database provides nationally representative estimates based on standardized protocols for data collection. Consequently, the current findings can be generalized widely. However, it is important to note that our study has several limitations. First, the study only included the general population of the United States from NHANES 2013–2014 due to year limitations. Second, we could not analyze individual details regarding diet and medication, which might affect calcification. Third, the self-reported confounders may be biased due to the self-reported questionnaire. Fourth, in Tables 3 and 2, several ORs and confidence intervals (CIs) appear small or borderline significant. This may reflect limited statistical power when adjusting for numerous covariates. In the future, we can enhance the statistical power by increasing the sample size, optimizing the selection of covariates and reasonably setting the significance level, thereby detecting the true effect more accurately. Finally, as a cross-sectional study, conclusions were limited to associations rather than causality. Future research will need to be conducted with a forward-looking perspective to confirm causal relationships.

## 5. Conclusion

In the general U.S. population, we observed a U-shaped relationship between serum total bilirubin levels and the risk of AAC and SAAC. Maintaining optimal serum total bilirubin levels may be crucial for the prevention of AAC. These findings provide a foundation for future research exploring changes in oxidative stress markers and the underlying mechanisms of AAC.

## Figures and Tables

**Table 1:** Study population data according to total bilirubin quartiles.

Total bilirubin	Total (3016)	Q1 (1245)	Q2 (540)	Q3 (714)	Q4 (517)	*p*-Value
Age (years)	57.43 ± 0.29	57.09 ± 0.36	57.25 ± 0.41	58.09 ± 0.70	57.42 ± 0.56	0.635
Sex (%)	—	—	—	—	—	<0.001
Male	1454 (48.2%)	456 (15.1%)	253 (8.4%)	402 (13.3%)	343 (11.4%)	—
Female	1562 (51.8%)	789 (26.2%)	287 (9.5%)	312 (10.3%)	174 (5.8%)	—
Race (%)	—	—	—	—	—	0.106
Mexican American	400 (13.3%)	164 (5.4%)	74 (2.5%)	90 (3.0%)	72 (2.4%)	—
Other Hispanic	286 (9.5%)	128 (4.2%)	48 (1.6%)	70 (2.3%)	40 (1.3%)	—
Non-Hispanic Black	576 (19.1%)	265 (8.8%)	109 (3.6%)	117 (3.9%)	85 (2.8%)	—
Non-Hispanic White	1338 (44.4%)	534 (17.7%)	238 (7.9%)	328 (10.9%)	238 (7.9%)	—
Other race	416 (13.8%)	154 (5.1%)	71 (2.4%)	109 (3.6%)	82 (2.7%)	—
Family PIR	3.16 ± 0.11	2.93 ± 0.12	3.15 ± 0.11	3.33 ± 0.17	3.46 ± 0.12	<0.001
Education level (%)	—	—	—	—	—	0.002
Less than high school	689 (22.8%)	309 (10.2%)	121 (4.0%)	162 (5.4%)	97 (3.2%)	—
High school	684 (22.7%)	291 (9.6%)	133 (4.4%)	154 (5.1%)	106 (3.5%)	—
More than high school	1643 (54.5%)	645 (21.4%)	286 (9.5%)	398 (13.2%)	314 (10.4%)	—
Marital status (%)	—	—	—	—	—	0.001
Having a partner	1936 (64.2%)	733 (24.3%)	334 (11.1%)	491 (16.3%)	378 (12.5%)	—
No partner	841 (27.9%)	396 (13.1%)	157 (5.2%)	181 (6.0%)	107 (3.5%)	—
Unmarried	239 (7.9%)	116 (3.8%)	49 (1.6%)	42 (1.4%)	32 (1.1%)	—
Hypertension (%)	—	—	—	—	—	0.885
No	1394 (46.2%)	559 (18.5%)	247 (8.2%)	335 (11.1%)	253 (8.4%)	—
Yes	1622 (53.8%)	686 (22.7%)	293 (9.7%)	379 (12.6%)	264 (8.8%)	—
DM (%)	—	—	—	—	—	0.487
No	2304 (76.4%)	936 (31.0%)	405 (13.4%)	560 (18.6%)	403 (16.0%)	—
Yes	712 (23.6%)	309 (10.2%)	135 (4.5%)	154 (5.1%)	114 (3.8%)	—
Smoker (%)	—	—	—	—	—	<0.001
No	1625 (53.9%)	651 (21.6%)	286 (9.5%)	382 (12.7%)	306 (10.1%)	—
Former	836 (27.7%)	315 (10.4%)	137 (4.5%)	222 (7.4%)	162 (5.4%)	—
Now	555 (18.4%)	279 (9.3%)	117 (3.9%)	110 (3.6%)	49 (1.6%)	—
Alcohol user (%)	—	—	—	—	—	0.028
Never	448 (14.9%)	218 (7.2%)	68 (2.3%)	101 (3.3%)	61 (2.0%)	—
Former	609 (20.2%)	271 (9.0%)	109 (3.6%)	130 (4.3%)	99 (3.3%)	—
Mild	1124 (37.3%)	422 (14.0%)	203 (6.7%)	278 (9.2%)	221 (7.3%)	—
Moderate	433 (14.4%)	163 (5.4%)	87 (2.9%)	118 (3.9%)	65 (2.2%)	—
Heavy	402 (13.3%)	171 (5.7%)	73 (2.4%)	87 (2.9%)	71 (2.4%)	—
CHD (%)	—	—	—	—	—	0.106
No	2856 (94.7%)	1173 (38.9%)	518 (17.2%)	681 (22.6%)	484 (16.0%)	—
Yes	160 (5.3%)	72 (2.4%)	22 (0.7%)	33 (1.1%)	33 (1.1%)	—
CHF (%)	—	—	—	—	—	0.184
No	2913 (96.6%)	1206 (40.0%)	526 (17.4%)	691 (22.9%)	490 (16.2%)	—
Yes	103 (3.4%)	39 (1.3%)	14 (0.5%)	23 (0.8%)	27 (0.9%)	—
Angina pectoris (%)	—	—	—	—	—	0.385
No	2919 (96.8%)	1202 (39.9%)	526 (17.4%)	692 (22.9%)	499 (16.5%)	—
Yes	97 (3.2%)	43 (1.4%)	14 (0.5%)	22 (0.7%)	18 (0.6%)	—
Heart attack (%)	—	—	—	—	—	0.196
No	2864 (95.0%)	1181 (39.2%)	511 (16.9%)	688 (22.8%)	484 (16.0%)	—
Yes	152 (5.0%)	64 (2.1%)	29 (1.0%)	26 (0.9%)	33 (1.1%)	—
Stroke (%)	—	—	—	—	—	0.572
No	2886 (95.7%)	1188 (39.4%)	518 (17.2%)	692 (22.9%)	488 (16.2%)	—
Yes	130 (4.3%)	57 (1.9%)	22 (0.7%)	22 (0.7%)	29 (1.0%)	—
Hyperlipidemia (%)	—	—	—	—	—	0.468
No	690 (22.9%)	259 (8.6%)	129 (4.3%)	173 (5.7%)	129 (4.3%)	—
Yes	2326 (77.1%)	986 (32.7%)	411 (13.6%)	541 (17.9%)	388 (12.9%)	—
CKD (%)	—	—	—	—	—	0.785
No	2371 (78.6%)	967 (32.1%)	414 (13.7%)	570 (18.9%)	420 (13.9%)	—
Yes	645 (21.4%)	278 (9.2%)	126 (4.2%)	144 (4.8%)	97 (3.2%)	—
Statins drugs	—	—	—	—	—	0.964
No	2168 (71.9%)	896 (29.7%)	388 (12.9%)	519 (17.2%)	365 (12.1%)	—
Yes	848 (28.1%)	349 (11.6%)	152 (5.0%)	195 (6.5%)	152 (5.0%)	—
BMI (kg/m^2)^	28.54 ± 0.17	29.35 ± 0.36	28.57 ± 0.34	27.64 ± 0.27	27.97 ± 0.22	0.896
Waist circumference (cm)	99.84 ± 0.33	101.06 ± 0.78	99.55 ± 0.73	98.38 ± 0.64	99.53 ± 0.69	0.079
Mean energy intake (kcal/day)	2023.55 ± 24.40	2026.63 ± 38.70	1980.97 ± 46.32	2030.25 ± 47.01	2050.09 ± 59.50	0.653
Dietary calcium intake (mg)	933.14 ± 8.51	931.10 ± 19.86	940.12 ± 22.78	917.07 ± 22.13	953.62 ± 36.26	0.889
Dietary phosphorus intake (mg)	1354.91 ± 13.40	1327.90 ± 21.88	1351.16 ± 24.20	1362.64 ± 33.87	1407.95 ± 37.44	0.262
WBC (1000 cells/μL)	7.19 ± 0.06	7.59 ± 0.08	7.20 ± 0.11	6.98 ± 0.12	6.62 ± 0.09	<0.001
Neu (1000 cells/μL)	4.31 ± 0.04	4.53 ± 0.07	4.33 ± 0.09	4.16 ± 0.09	4.01 ± 0.06	<0.001
Lym (1000 cells/μL)	2.05 ± 0.02	2.19 ± 0.02	2.02 ± 0.04	1.99 ± 0.03	1.84 ± 0.03	<0.001
Monocyte (1000 cells/μL)	0.59 ± 0.01	0.60 ± 0.01	0.59 ± 0.01	0.58 ± 0.01	0.55 ± 0.01	0.017
MCV/fL	90.41 ± 0.19	89.65 ± 0.18	90.85 ± 0.36	90.97 ± 0.35	90.87 ± 0.33	0.001
RDW (%)	13.64 ± 0.03	13.80 ± 0.08	13.59 ± 0.05	13.53 ± 0.06	13.49 ± 0.04	0.048
Platelet (10^9^/L)	230.84 ± 1.60	242.21 ± 2.54	235.39 ± 2.83	221.72 ± 2.24	213.88 ± 3.25	<0.001
Mean platelet volume (fL)	8.42 ± 0.03	8.42 ± 0.06	8.42 ± 0.06	8.42 ± 0.05	8.44 ± 0.05	0.990
NLR	2.31 ± 0.02	2.28 ± 0.05	2.33 ± 0.07	2.30 ± 0.05	2.36 ± 0.05	0.601
PLR	123.82 ± 1.23	121.82 ± 1.35	128.10 ± 3.29	122.57 ± 1.50	125.73 ± 2.30	0.033
NAR	0.10 ± 0.00	0.11 ± 0.00	0.10 ± 0.00	0.10 ± 0.00	0.09 ± 0.00	<0.001
SII index	534.64 ± 8.01	555.35 ± 14.64	551.79 ± 21.29	511.57 ± 10.91	504.08 ± 13.16	0.002
SIRI	1.39 ± 0.02	1.40 ± 0.03	1.41 ± 0.05	1.39 ± 0.06	1.34 ± 0.04	0.439
Alk (U/L)	65.77 ± 0.76	67.20 ± 0.92	66.87 ± 1.47	63.06 ± 1.21	65.35 ± 1.36	0.064
Alb (g/L)	42.51 ± 0.10	41.86 ± 0.12	42.36 ± 0.13	43.07 ± 0.11	43.30 ± 0.20	<0.001
GGT (IU/L)	28.68 ± 1.19	26.10 ± 1.12	29.79 ± 2.67	29.84 ± 2.08	31.63 ± 2.61	0.024
Serum iron (μg/mL)	84.74 ± 1.11	70.35 ± 1.20	83.19 ± 1.09	95.25 ± 1.81	103.40 ± 2.64	<0.001
Calcium (mg/dL)	9.45 ± 0.01	9.44 ± 0.02	9.45 ± 0.02	9.46 ± 0.02	9.48 ± 0.02	0.345
Phosphorus (mg/dL)	3.80 ± 0.02	3.89 ± 0.02	3.77 ± 0.03	3.75 ± 0.02	3.69 ± 0.03	<0.001
FBG (mg/dL)	108.84 ± 0.86	110.49 ± 1.27	110.05 ± 2.56	107.32 ± 1.92	106.09 ± 1.72	0.326
TC (mg/dL)	195.62 ± 0.59	195.89 ± 1.58	193.16 ± 1.53	197.85 ± 1.55	194.29 ± 2.63	0.143
TG (mg/dL)	144.46 ± 2.80	151.56 ± 3.92	145.80 ± 6.75	140.98 ± 7.01	132.20 ± 5.58	0.029
HDL (mg/dL)	54.72 ± 0.36	53.70 ± 0.67	53.69 ± 0.94	56.44 ± 0.64	55.59 ± 1.20	0.025
BUN (mg/dL)	14.23 ± 0.11	14.17 ± 0.23	14.16 ± 0.24	14.46 ± 0.24	14.09 ± 0.25	0.626
UA (mg/dL)	5.41 ± 0.03	5.21 ± 0.07	5.33 ± 0.08	5.59 ± 0.08	5.68 ± 0.09	0.002
Scr (mg/dL)	0.93 ± 0.01	0.89 ± 0.01	0.93 ± 0.02	0.97 ± 0.02	0.94 ± 0.01	<0.001
eGFR (mL/min/1.73 m^2^)	84.29 ± 0.48	85.28 ± 0.82	84.26 ± 1.10	82.30 ± 0.76	84.93 ± 1.01	0.056
Calcification score	2.47 ± 0.10	2.45 ± 0.11	2.43 ± 0.27	2.54 ± 0.23	2.49 ± 0.20	0.977
AAC (%)	—	—	—	—	—	0.918
No	2107 (69.9%)	885 (29.3%)	385 (12.8%)	494 (16.4%)	343 (11.4%)	—
Yes	909 (30.1%)	360 (11.9%)	155 (5.1%)	220 (7.3%)	174 (5.8%)	—
SAAC (%)	—	—	—	—	—	0.979
No	2689 (89.2%)	1118 (37.1%)	479 (15.9%)	635 (21.1%)	457 (15.2%)	—
Yes	327 (10.8%)	127 (4.2%)	61 (2.0%)	79 (2.6%)	60 (2.0%)	—

*Note:* Q1, 0.10–0.50 g/dL; Q2, 0.51–0.60 g/dL; Q3, 0.61–0.80 g/dL; Q4, 0.81–2.20 g/dL; HbA1c, glycosylated hemoglobin.

Abbreviations: AAC, abdominal aortic calcification; Alb, albumin; Alk, alkaline phosphatase; BMI, body mass index; BUN, blood urea nitrogen; CHD, coronary heart disease; CHF, congestive heart failure; CKD, chronic kidney diseases; DM, diabetes mellitus; eGFR, estimated glomerular filtration rate; FBG, fast glucose; GGT, gamma glutamyl transferase; HDL, cholesterol; high-density lipoprotein-cholesterol; Lym, lymphocyte; MCV, mean cell volume; NAR, neutrophil-to-albumin ratio; Neu, neutrophil; NLR, neutrophil-to-lymphocyte ratio; PLR, platelet-to-lymphocyte ratio; RDW, red cell distribution width; SAAC, severe abdominal aortic calcification; Scr, serum creatinine; SII index, systemic immune inflammation index; SIRI, system inflammation response index; TC, total cholesterol; TG, triglycerides; UA, uric acid; WBC, white blood cells.

**Table 2:** Adjusted ORs for associations between total bilirubin and risk of SAAC.

Total bilirubin	Model 1	Model 2	Model 3
	OR (95% CI)	OR (95% CI)	OR (95% CI)
Q1	Ref.	Ref.	Ref.
Q2	0.71 (0.55, 0.97)*⁣*^*∗*^	0.80 (0.61, 1.13)	0.78 (0.58, 1.21)
Q3	0.98 (0.67, 1.42)	1.06 (0.73, 1.43)	1.12 (0.77, 1.65)
Q4	1.12 (0.81, 1.47)	1.29 (0.91, 1.76)	1.28 (0.87, 1.77)
*p* for trend	0.723	0.499	0.593

*Note:* Q1, 0.10–0.50 g/dL; Q2, 0.51–0.60 g/dL; Q3, 0.61–0.80 g/dL; Q4, 0.81–0.71 g/dL. Model 1: age and sex. Model 2: Model 1 variables plus race/ethnicity, education level, marital status, family poverty-income ratio, the complication of hypertension, and diabetes mellitus, smoker, alcohol user. Model 3 was adjusted for Model 2 variables plus body mass index, waist circumference, the complication of coronary heart disease, congestive heart failure, angina pectoris, heart attack, and stroke, mean energy intake, calcium intake and phosphorus intake, fast glucose, glycosylated hemoglobin, total cholesterol and triglyceride, high-density lipoprotein-cholesterol, blood urea nitrogen, serum uric acid, serum creatinine, estimated glomerular filtration rate, serum calcium, phosphorus, lymphocyte, monocyte, mean cell volume, red cell distribution width, platelet, mean platelet volume, neutrophil-to-lymphocyte ratio, platelet-to-lymphocyte ratio, systemic immune inflammation index, system inflammation response index, albumin, gamma glutamyl transferase, alkaline phosphatase, and serum iron.

Abbreviations: CI, confidence interval; OR, odd ratio; SAAC, severe abdominal aortic calcification.

*⁣* ^*∗*^ *p*  < 0.05

**Table 3:** Adjusted ORs for associations between total bilirubin and risk of AAC.

Total bilirubin	Model 1	Model 2	Model 3
	OR (95% CI)	OR (95% CI)	OR (95% CI)
Q1	Ref.	Ref.	Ref.
Q2	0.72 (0.52, 0.97)*⁣*^*∗*^	0.73 (0.52, 0.99)*⁣*^*∗*^	0.71 (0.61, 0.98)*⁣*^*∗*^
Q3	1.09 (0.90, 1.34)	1.15 (0.94, 1.42)	1.11 (0.90, 1.38)
Q4	1.27 (1.01, 1.59)*⁣*^*∗*^	1.40 (1.11, 1.78)*⁣*^*∗∗*^	1.36 (1.07, 1.73)*⁣*^*∗*^
*p* for trend	0.041	0.015	0.035

*Note:* Q1, 0.10–0.50 g/dL; Q2, 0.51–0.60 g/dL; Q3, 0.61–0.80 g/dL; Q4, 0.81–0.71 g/dL. Model 1: age and sex. Model 2: Model 1 variables plus race/ethnicity, education level, marital status, family poverty-income ratio, the complication of hypertension, and diabetes mellitus, smoker, alcohol user; Model 3 was adjusted for Model 2 variables plus body mass index, waist circumference, the complication of coronary heart disease, congestive heart failure, angina pectoris, heart attack, and stroke, mean energy intake, calcium intake, and phosphorus intake, fast glucose, glycosylated hemoglobin, total cholesterol, and triglyceride, high-density lipoprotein-cholesterol, blood urea nitrogen, serum uric acid, serum creatinine, estimated glomerular filtration rate, serum calcium, phosphorus, lymphocyte, monocyte, mean cell volume, red cell distribution width, platelet, mean platelet volume, neutrophil-to-lymphocyte ratio, platelet-to-lymphocyte ratio, systemic immune inflammation index, system inflammation response index, albumin, gamma glutamyl transferase, alkaline phosphatase, and serum iron.

Abbreviations: AAC, abdominal aortic calcification; CI, confidence interval; OR, odd ratio.

*⁣* ^*∗*^ *p*  < 0.05.

*⁣* ^*∗∗*^ *p*  < 0.01.

## Data Availability

The survey data are available for data consumers and researchers all across the globe on the internet (https://www.cdc.gov/nchs/nhanes/).

## Supporting Information

Supporting Information 1 Table S3. Multicollinearity tests based on Model 3 in AAC.

Supporting Information 2 Table S4. Multicollinearity tests based on Model 3 in SAAC.

Supporting Information 3 Table S1. Subgroups analysis for the associations of total bilirubin with the risk of SAAC.

Supporting Information 4 Table S2. Subgroups analysis for the associations of total bilirubin with the risk of AAC.

Supporting Information 5 Figure S1. Restricted cubic spline curve for the association of serum total bilirubin level with the risk of AAC stratified by (A) age, (B) sex, (C) hypertension, (D) DM, and (E) BMI. AAC, abdominal aortic calcification; BMI, body mass index; DM, diabetes mellitus.

Supporting Information 6 Figure S2. Restricted cubic spline curve for the association of serum total bilirubin level with the risk of SAAC stratified by (A) age, (B) sex, (C) hypertension, (D) DM, and (E) BMI. BMI, body mass index; DM, diabetes mellitus; SAAC, severe abdominal aortic calcification.

Supporting Information 7 Figure S3. Mediation analysis of lipid profiles on the interaction between total bilirubin and AAC. (A) mediation models of TC, total bilirubin, and AAC: Direct effect (TE = −0.05769; *p*=0.720) of total bilirubin (exposure) toward AAC (outcome), and TC mediation proportion is −2.78%; indirect effect (IE = 0.01173; *p*=0.170) of total bilirubin (exposure) toward TC (mediator) and effect AAC (DE = −0.06942; *p*=0.670), from TC (mediator) toward AAC (outcome). (B) Mediation models of TG, total bilirubin, and AAC: direct effect (TE = −0.0512; *p*=0.740) of total bilirubin (exposure) toward AAC (outcome), and TG mediation proportion is 2.56%; indirect effect (IE = −0.0112; *p*=0.130) of total bilirubin (exposure) toward TG (mediator) and effect AAC (DE = −0.0400; *p*=0.800), from TG (mediator) toward AAC (outcome). (C) Mediation models of HDL-cholesterol, total bilirubin, and AAC: Direct effect (TE = −0.0562; *p*=0.732) of total bilirubin (exposure) toward AAC (outcome), and HDL-cholesterol mediation proportion is 8.69%; indirect effect (IE = −0.0281; *p*=0.048) of total bilirubin (exposure) toward HDL-cholesterol (mediator) and effect AAC (DE = −0.0281; *p*=0.852), from HDL-cholesterol (mediator) toward AAC (outcome). (D) Mediation models of LDL-cholesterol, total bilirubin, and AAC: direct effect (TE = −0.04271; *p*=0.800) of total bilirubin (exposure) toward AAC (outcome), and LDL-cholesterol mediation proportion is −1.67%; indirect effect (IE = 0.01556; *p*=0.220) of total bilirubin (exposure) toward LDL-cholesterol (mediator) and effect AAC (DE = −0.05827; *p*=0.730), from LDL-cholesterol (mediator) toward AAC (outcome). AAC, abdominal aortic calcification; high density lipoprotein-cholesterol; LDL-cholesterol, low density lipoprotein-cholesterol; TC, total cholesterol; TG, triglycerides.

Supporting Information 8 Figure S4. Mediation analysis of lipid profiles on the interaction between total bilirubin and SAAC. (A) Mediation models of TC, total bilirubin, and SAAC: Direct effect (TE = −0.010587; *p*=0.490) of total bilirubin (exposure) toward SAAC (outcome), and TC mediation proportion is −3.75%; indirect effect (IE = 0.001136; *p*=0.170) of total bilirubin (exposure) toward TC (mediator) and effect SAAC (DE = −0.011723; *p*=0.450), from TC (mediator) toward SAAC (outcome). (B) Mediation models of TG, total bilirubin, and SAAC: Direct effect (TE = −0.010328; *p*=0.480) of total bilirubin (exposure) toward SAAC (outcome), and TG mediation proportion is 2.66%; indirect effect (IE = −0.000742; *p*=0.180) of total bilirubin (exposure) toward TG (mediator) and effect SAAC (DE = −0.011070; *p*=0.450), from TG (mediator) toward SAAC (outcome). (C) Mediation models of HDL-cholesterol, total bilirubin, and SAAC: Direct effect (TE = −0.00879; *p*=0.570) of total bilirubin (exposure) toward SAAC (outcome), and HDL-cholesterol mediation proportion is 9.00%; indirect effect (IE = −0.00217; *p*=0.058) of total bilirubin (exposure) toward HDL-cholesterol (mediator) and effect SAAC (DE = −0.01095; *p*=0.468), from HDL-cholesterol (mediator) toward SAAC (outcome). (D) Mediation models of LDL-cholesterol, total bilirubin, and SAAC: Direct effect (TE = −0.011701; *p*=0.460) of total bilirubin (exposure) toward SAAC (outcome), and LDL-cholesterol mediation proportion is −4.90%; indirect effect (IE = 0.001404; *p*=0.160) of total bilirubin (exposure) toward LDL-cholesterol (mediator) and effect SAAC (DE = −0.010297; *p*=0.500), from LDL-cholesterol (mediator) toward SAAC (outcome). HDL-cholesterol, high density lipoprotein-cholesterol; LDL-cholesterol, low density lipoprotein-cholesterol; SAAC, severe abdominal aortic calcification; TC, total cholesterol; TG, triglycerides.

## References

[1] Ngai D., Lino M., Bendeck M. P. (2018). Cell-Matrix Interactions and Matricrine Signaling in the Pathogenesis of Vascular Calcification. *Frontiers in Cardiovascular Medicine*.

[2] Wu X. H., Chen X.-Y., Wang L. J., Wong K. S. (2016). Intracranial Artery Calcification and Its Clinical Significance. *Journal of Clinical Neurology*.

[3] Wilson P. W., Kauppila L. I., O’Donnell C. J. (2001). Abdominal Aortic Calcific Deposits Are an Important Predictor of Vascular Morbidity and Mortality. *Circulation*.

[4] Itani Y., Watanabe S., Masuda Y. (2006). Relationship Between Aortic Calcification and Stroke in a Mass Screening Program Using a Mobile Helical Computed Tomography Unit. *Circulation Journal*.

[5] Rodondi N., Taylor B. C., Bauer D. C. (2007). Association Between Aortic Calcification and Total and Cardiovascular Mortality in Older Women. *Journal of Internal Medicine*.

[6] Bastos Gonçalves F., Voûte M. T., Hoeks S. E. (2012). Calcification of the Abdominal Aorta as an Independent Predictor of Cardiovascular Events: A Meta-Analysis. *Heart*.

[7] Chuang T.-L., Li Y.-D., Hsiao F.-T., Chuang M.-H., Wang Y.-F. (2017). FRAX Fracture Risks Are Associated With Coronary Artery Calcification Score. *Disease Markers*.

[8] Khansari N., Shakiba Y., Mahmoudi M. (2009). Chronic Inflammation and Oxidative Stress as a Major cause of Age-Related Diseases and Cancer. *Recent Patents on Inflammation & Allergy Drug Discovery*.

[9] Biswas S. K. (2016). Does the Interdependence Between Oxidative Stress and Inflammation Explain the Antioxidant Paradox?. *Oxidative Medicine and Cellular Longevity*.

[10] Stranges S., Dorn J. M., Muti P. (2004). Body Fat Distribution, Relative Weight, and Liver Enzyme Levels: A Population-Based Study. *Hepatology*.

[11] Nakanishi N., Yoshida H., Nakamura K., Suzuki K., Tatara K. (2001). Predictors for Development of Hyperuricemia: An 8-Year Longitudinal Study in Middle-Aged Japanese Men. *Metabolism-Clinical and Experimental*.

[12] Ford E. S. (1999). Body Mass Index, Diabetes, and C-Reactive Protein Among U.S. Adults. *Diabetes Care*.

[13] Targher G. (2010). Elevated Serum Gamma-Glutamyltransferase Activity Is Associated With Increased Risk of Mortality, Incident Type 2 Diabetes, Cardiovascular Events, Chronic Kidney Disease and Cancer — a Narrative Review. *Clinical Chemistry and Laboratory Medicine*.

[14] Kaptoge S., Di Angelantonio E., Lowe G. (2010). C-Reactive Protein Concentration and Risk of Coronary Heart Disease, Stroke, and Mortality: An Individual Participant Meta-Analysis. *The Lancet*.

[15] Kim S. Y., Guevara J. P., Kim K. M., Choi H. K., Heitjan D. F., Albert D. A. (2010). Hyperuricemia and Coronary Heart Disease: A Systematic Review and Meta-Analysis. *Arthritis Care & Research*.

[16] Inoguchi T., Sonoda N., Maeda Y. (2016). Bilirubin as an Important Physiological Modulator of Oxidative Stress and Chronic Inflammation in Metabolic Syndrome and Diabetes: A New Aspect on Old Molecule. *Diabetology International*.

[17] Lapenna D., Ciofani G., Pierdomenico S. D., Giamberardino M. A., Ucchino S., Davì G. (2018). Association of Serum Bilirubin With Oxidant Damage of Human Atherosclerotic Plaques and the Severity of Atherosclerosis. *Clinical and Experimental Medicine*.

[18] Li C., Wu W., Song Y., Xu S., Wu X. (2022). The Nonlinear Relationship Between Total Bilirubin and Coronary Heart Disease: A Dose-Response Meta-Analysis. *Frontiers in Cardiovascular Medicine*.

[19] Ahluwalia N., Dwyer J., Terry A., Moshfegh A., Johnson C. (2016). Update on NHANES Dietary Data: Focus on Collection, Release, Analytical Considerations, and Uses to Inform Public Policy. *Advances in Nutrition*.

[20] Zipf G., Chiappa M., Porter K. S., Ostchega Y., Lewis B. G., Dostal J. (2013). National Health and Nutrition Examination Survey: Plan and Operations. *Vital and Health Statistics*.

[21] Yin Y., Wu H., Lei F. (2023). Relationship Between Novel Anthropometric Indices and the Prevalence of Abdominal Aortic Calcification: A Large Cross-Sectional Study. *Reviews in Cardiovascular Medicine*.

[22] Sang T., Gao F., Lu X. (2024). Associations of Oxidative Stress Markers With the Prevalence of Sarcopenia in the United States General Population. *Clinics*.

[23] Hu B., Yang X. R., Xu Y. (2014). Systemic Immune-Inflammation Index Predicts Prognosis of Patients After Curative Resection for Hepatocellular Carcinoma. *Clinical Cancer Research*.

[24] Kauppila L. I., Polak J. F., Cupples L. A., Hannan M. T., Kiel D. P., Wilson P. W. F. (1997). New Indices to Classify Location, Severity and Progression of Calcific Lesions in the Abdominal Aorta: A 25-Year Follow-up Study. *Atherosclerosis*.

[25] Schousboe J. T., Wilson K. E., Hangartner T. N. (2007). Detection of Aortic Calcification During Vertebral Fracture Assessment (VFA) Compared to Digital Radiography. *PLoS ONE*.

[26] Wei R., Zhang Y., Huang M., Piao H., Gu Z., Zhu C. (2024). Associations Between Bone Mineral Density and Abdominal Aortic Calcification: Results of a Nationwide Survey. *Nutrition, Metabolism and Cardiovascular Diseases*.

[27] Chen W., Eisenberg R., Mowrey W. B. (2020). Association Between Dietary Zinc Intake and Abdominal Aortic Calcification in US Adults. *Nephrology Dialysis Transplantation*.

[28] Qin Z., Chang K., Liao R., Jiang L., Yang Q., Su B. (2021). Greater Dietary Inflammatory Potential Is Associated With Higher Likelihood of Abdominal Aortic Calcification. *Frontiers in Cardiovascular Medicine*.

[29] Górriz J. L., Molina P., Cerverón M. J. (2015). Vascular Calcification in Patients With Nondialysis CKD Over 3 Years. *Clinical Journal of the American Society of Nephrology*.

[30] Liao Y. (2021). Ride-Sourcing Compared to Its Public-Transit Alternative Using Big Trip Data. *Journal of Transport Geography*.

[31] Li M., Zhang W., Zhang M. (2024). Nonlinear Relationship Between Untraditional Lipid Parameters and the Risk of Prediabetes: A Large Retrospective Study Based on Chinese Adults. *Cardiovascular Diabetology*.

[32] Lee S. J., Lee I. K., Jeon J. H. (2020). Vascular Calcification—New Insights Into Its Mechanism. *International Journal of Molecular Sciences*.

[33] Zhou Y., Hellberg M., Hellmark T., Höglund P., Clyne N. (2020). Twelve Months of Exercise Training Did Not Halt Abdominal Aortic Calcification in Patients With CKD – a Sub-Study of RENEXC-a Randomized Controlled Trial. *BMC Nephrology*.

[34] Sun H., Zhang F., Xu Y. (2019). Salusin-*β* Promotes Vascular Calcification via Nicotinamide Adenine Dinucleotide Phosphate/Reactive Oxygen Species-Mediated Klotho Downregulation. *Antioxidants & Redox Signaling*.

[35] Kang S. J., Kim D., Park H. E. (2013). Elevated Serum Bilirubin Levels Are Inversely Associated With Coronary Artery Atherosclerosis. *Atherosclerosis*.

[36] Zhang Z-Yun, Bian L-Qin, Kim S-Joo, Zhou C-Cun, Choi Y-Ho (2012). Inverse Relation of Total Serum Bilirubin to Coronary Artery Calcification Score Detected by Multidetector Computed Tomography in Males. *Clinical Cardiology*.

[37] Pae H.-O., Oh G.-S., Lee B.-S., Rim J.-S., Kim Y.-M., Chung H.-T. (2006). 3-Hydroxyanthranilic Acid, One of L-Tryptophan Metabolites, Inhibits Monocyte Chemoattractant Protein-1 Secretion and Vascular Cell Adhesion Molecule-1 Expression via Heme Oxygenase-1 Induction in Human Umbilical Vein Endothelial Cells. *Atherosclerosis*.

[38] Vitek L., Hinds T. D., Stec D. E., Tiribelli C. (2023). The Physiology of Bilirubin: Health and Disease Equilibrium. *Trends in Molecular Medicine*.

[39] Balogh E., Chowdhury A., Ababneh H., Csiki D. M., Tóth A., Jeney V. (2021). Heme-Mediated Activation of the Nrf2/HO-1 Axis Attenuates Calcification of Valve Interstitial Cells. *Biomedicines*.

[40] Nilsen D. W. T., Myhre P. L., Solheim S. (2023). Total Bilirubin Yields Prognostic Information Following a Myocardial Infarction in the Elderly. *Antioxidants*.

[41] Gullu H., Erdogan D., Tok D. (2005). High Serum Bilirubin Concentrations Preserve Coronary Flow Reserve and Coronary Microvascular Functions. *Arteriosclerosis, Thrombosis, and Vascular Biology*.

[42] Akboga M. K., Canpolat U., Sahinarslan A. (2015). Association of Serum Total Bilirubin Level With Severity of Coronary Atherosclerosis Is Linked to Systemic Inflammation. *Atherosclerosis*.

[43] Wang J., Zhang X., Zhang Z. (2020). Baseline Serum Bilirubin and Risk of First Stroke in Hypertensive Patients. *Journal of the American Heart Association*.

[44] Kang S. J., Lee C., Kruzliak P. (2014). Effects of Serum Bilirubin on Atherosclerotic Processes. *Annals of Medicine*.

[45] Higashi Y., Maruhashi T., Noma K., Kihara Y. (2014). Oxidative Stress and Endothelial Dysfunction: Clinical Evidence and Therapeutic Implications. *Trends in Cardiovascular Medicine*.

[46] Chen J., Lin Y., Teng Z., Lin Z., Li J., Zeng Q. (2024). Association Between Methylmalonic Acid and Abdominal Aortic Calcification in Adults: A Cross-Sectional Study. *Cardiovascular Innovations and Applications*.

[47] Wang F., Zheng J. (2022). Association Between Serum Alpha-Klotho and Severe Abdominal Aortic Calcification Among Civilians in the United States. *Nutrition, Metabolism and Cardiovascular Diseases*.

[48] Wang F., Zheng J. (2023). Clinical Relevance of Uric Tobacco-Specific Nitrosamine and Severe Abdominal Aortic Calcification in a National Survey of the United States. *Cardiovascular Innovations and Applications*.

[49] Li W., Wang Z., Li M., Xie J., Gong J., Liu N. (2023). Association Between a Body Shape Index and Abdominal Aortic Calcification in General Population: A Cross-Sectional Study. *Frontiers in Cardiovascular Medicine*.

[50] Ivanov S. M., Lagunin A. A., Filimonov D. A., Poroikov V. V. (2022). Relationships Between the Structure and Severe Drug-Induced Liver Injury for Low, Medium, and High Doses of Drugs. *Chemical Research in Toxicology*.

[51] Gastaldelli A., Stefan N., Häring H.-U. (2021). Liver-Targeting Drugs and Their Effect on Blood Glucose and Hepatic Lipids. *Diabetologia*.

[52] Keane J. T., Elangovan H., Stokes R. A., Gunton J. E. (2018). Vitamin D and the Liver—Correlation or Cause?. *Nutrients*.

[53] Pop T. L., Sîrbe C., Benţa G., Mititelu A., Grama A. (2022). The Role of Vitamin D and Vitamin D Binding Protein in Chronic Liver Diseases. *International Journal of Molecular Sciences*.

[54] Hammer F., Buehling S. S., Masyout J. (2021). Protective Effects of Spironolactone on Vascular Calcification in Chronic Kidney Disease. *Biochemical and Biophysical Research Communications*.

[55] Laurain A., Rubera I., Razzouk-Cadet M. (2022). Arterial Calcifications in Patients With Liver Cirrhosis Are Linked to Hepatic Deficiency of Pyrophosphate Production Restored by Liver Transplantation. *Biomedicines*.

[56] Jovanovich A., Isakova T., Block G. (2018). Deoxycholic Acid, a Metabolite of Circulating Bile Acids, and Coronary Artery Vascular Calcification in CKD. *American Journal of Kidney Diseases: the Official Journal of the National Kidney Foundation*.

[57] Woodward H. J., Zhu D., Hadoke P. W. F., MacRae V. E. (2021). Regulatory Role of Sex Hormones in Cardiovascular Calcification. *International Journal of Molecular Sciences*.

[58] Demer L. L., Tintut Y. (2014). Inflammatory, Metabolic, and Genetic Mechanisms of Vascular Calcification. *Arteriosclerosis, Thrombosis, and Vascular Biology*.

[59] Chavel S. M., Taraszka K. S., Schaffer J. V., Lazova R., Schechner J. S. (2004). Calciphylaxis Associated With Acute, Reversible Renal Failure in the Setting of Alcoholic Cirrhosis. *Journal of the American Academy of Dermatology*.

