# Supplementary material for: Association of Serum Total Bilirubin Level With Abdominal Aortic Calcification: A Population-Based Cross-Sectional Study
Source: Mediators Inflamm. 2025 Jul 27;2025:5229580. doi: 10.1155/mi/5229580 (PMC12318627; doi:10.1155/mi/5229580)
Supplement: Supporting Information 1 — Table S3. Multicollinearity tests based on Model 3 in AAC. [file 5229580.f1.docx]

| **Supplementary Table 3.** Multicollinearity tests based on Model 3 in AAC. | |
| --- | --- |
| Variable | VIF |
| Age, years | 1.391 |
| Sex, % | 1.423 |
| Race, % | 1.286 |
| Family PIR | 1.221 |
| Education level, % | 1.309 |
| Marital status, % | 1.106 |
| Hypertension, % | 1.103 |
| DM, % | 1.309 |
| Smoker, % | 1.144 |
| Alcohol user, % | 1.503 |
| CHD, % | 1.219 |
| CHF, % | 1.092 |
| Angina pectoris, % | 1.114 |
| Heart attack, % | 1.161 |
| Stroke, % | 1.034 |
| Hyperlipidemia, % | 1.067 |
| CKD, % | 1.120 |
| Statins drugs | 1.224 |
| BMI, kg/m^2^ | 2.435 |
| Waist circumference, cm | 2.571 |
| Mean energy intake (kcal/day) | 2.040 |
| Dietary calcium intake, mg | 1.656 |
| Dietary phosphorus intake, mg | 2.470 |
| WBC, 1000 cells/uL | 9.994 |
| Neu, 1000 cells/uL | 13.791 |
| Lym, 1000 cells/uL | 4.150 |
| Monocyte, 1000 cells/uL | 2.211 |
| MCV, fL | 1.242 |
| RDW, % | 1.234 |
| Platelet, 10^9/L | 2.377 |
| Mean platelet volume, fL | 1.151 |
| NLR | 4.711 |
| PLR | 3.228 |
| NAR | 11.722 |
| SII index | 4.307 |
| SIRI | 3.295 |
| Alk, U/L | 1.141 |
| Alb, g/L | 2.516 |
| GGT, iu/L | 1.125 |
| Serum iron, ug/mL | 1.194 |
| Calcium, mg/dL | 1.186 |
| Phosphorus, mg/dL | 1.108 |
| FBG, mg/dL | 1.288 |
| TC, mg/dL | 1.451 |
| TG, mg/dL | 1.182 |
| HDL, mg/dL | 1.362 |
| BUN, mg/dL | 1.419 |
| UA, mg/dL | 1.273 |
| Scr, mg/dL | 1.515 |
| eGFR, ml/min/1.73m^2^ | 1.870 |

Abbreviations: AAC, abdominal aortic calcification; DM, diabetes mellitus; CHD, coronary heart disease; CHF, congestive heart failure; CKD, chronic kidney diseases; BMI, body mass index; WBC, white blood cells; Neu, neutrophil; Lym, lymphocyte; MCV, mean cell volume; RDW, red cell distribution width; NLR, neutrophil-to-lymphocyte ratio; PLR, platelet-to-lymphocyte ratio; NAR, neutrophil-to-albumin ratio; SII index, systemic immune inflammation index; SIRI, system inflammation response index; FBG, fast glucose; HbA1c, glycosylated hemoglobin; Alb, albumin; GGT, gamma glutamyl transferase; Alk, alkaline phosphatase; TC, total cholesterol; TG, triglycerides; HDL-cholesterol, high density lipoprotein-cholesterol; BUN, blood urea nitrogen; UA, uric acid; Scr, serum creatinine; eGFR, estimated glomerular filtration rate.
