# Supplementary material for: Association of Serum Total Bilirubin Level With Abdominal Aortic Calcification: A Population-Based Cross-Sectional Study
Source: Mediators Inflamm. 2025 Jul 27;2025:5229580. doi: 10.1155/mi/5229580 (PMC12318627; doi:10.1155/mi/5229580)
Supplement: Supporting Information 2 — Table S4. Multicollinearity tests based on Model 3 in SAAC. [file 5229580.f2.docx]

| **Supplementary Table 4.** Multicollinearity tests based on Model 3 in SAAC. | |
| --- | --- |
| Variable | VIF |
| Age, years | 1.479 |
| Sex, % | 1.577 |
| Race, % | 1.271 |
| Family PIR | 1.212 |
| Education level, % | 1.322 |
| Marital status, % | 1.143 |
| Hypertension, % | 1.077 |
| DM, % | 1.334 |
| Smoker, % | 1.186 |
| Alcohol user, % | 1.659 |
| CHD, % | 1.296 |
| CHF, % | 1.126 |
| Angina pectoris, % | 1.158 |
| Heart attack, % | 1.223 |
| Stroke, % | 1.052 |
| Hyperlipidemia, % | 1.251 |
| CKD, % | 1.290 |
| Statins drugs | 1.392 |
| BMI, kg/m^2^ | 2.372 |
| Waist circumference, cm | 2.519 |
| Mean energy intake (kcal/day) | 2.020 |
| Dietary calcium intake, mg | 1.657 |
| Dietary phosphorus intake, mg | 2.474 |
| WBC, 1000 cells/uL | 8.957 |
| Neu, 1000 cells/uL | 12.807 |
| Lym, 1000 cells/uL | 3.905 |
| Monocyte, 1000 cells/uL | 2.049 |
| MCV, fL | 1.243 |
| RDW, % | 1.239 |
| Platelet, 10^9/L | 2.317 |
| Mean platelet volume, fL | 1.148 |
| NLR | 4.861 |
| PLR | 3.217 |
| NAR | 11.317 |
| SII index | 4.691 |
| SIRI | 3.049 |
| Alk, U/L | 1.180 |
| Alb, g/L | 2.494 |
| GGT, iu/L | 1.145 |
| Serum iron, ug/mL | 1.221 |
| Calcium, mg/dL | 1.194 |
| Phosphorus, mg/dL | 1.137 |
| FBG, mg/dL | 1.265 |
| TC, mg/dL | 1.494 |
| TG, mg/dL | 1.387 |
| HDL, mg/dL | 1.549 |
| BUN, mg/dL | 1.505 |
| UA, mg/dL | 1.275 |
| Scr, mg/dL | 1.578 |
| eGFR, ml/min/1.73m^2^ | 2.007 |

Abbreviations: SAAC, severe abdominal aortic calcification; DM, diabetes mellitus; CHD, coronary heart disease; CHF, congestive heart failure; CKD, chronic kidney diseases; BMI, body mass index; WBC, white blood cells; Neu, neutrophil; Lym, lymphocyte; MCV, mean cell volume; RDW, red cell distribution width; NLR, neutrophil-to-lymphocyte ratio; PLR, platelet-to-lymphocyte ratio; NAR, neutrophil-to-albumin ratio; SII index, systemic immune inflammation index; SIRI, system inflammation response index; FBG, fast glucose; HbA1c, glycosylated hemoglobin; Alb, albumin; GGT, gamma glutamyl transferase; Alk, alkaline phosphatase; TC, total cholesterol; TG, triglycerides; HDL-cholesterol, high density lipoprotein-cholesterol; BUN, blood urea nitrogen; UA, uric acid; Scr, serum creatinine; eGFR, estimated glomerular filtration rate.
