# Supplementary material for: Association of Serum Total Bilirubin Level With Abdominal Aortic Calcification: A Population-Based Cross-Sectional Study
Source: Mediators Inflamm. 2025 Jul 27;2025:5229580. doi: 10.1155/mi/5229580 (PMC12318627; doi:10.1155/mi/5229580)
Supplement: Supporting Information 3 — Table S1. Subgroups analysis for the associations of total bilirubin with the risk of SAAC. [file 5229580.f3.docx]

| **Supplementary Table 1.** Subgroups analysis for the associations of total bilirubin with the risk of AAC. | | | | | | |
| --- | --- | --- | --- | --- | --- | --- |
| Total | Q1 | Q2 | Q3 | Q4 | *P* for trend | *P* for interaction |
| bilirubin | OR (95%CI) | OR (95%CI) | OR (95%CI) | OR (95%CI) |  |  |
| Age |  |  |  |  |  | 0.539 |
| < 60 | Ref. | 0.98 (0.66, 1.45) | 1.04 (0.72, 1.51) | 1.45 (0.98, 2.16) | 0.118 |  |
| ≥ 60 | Ref. | 0.90 (0.65, 1.27) | 1.08 (0.79, 1.48) | 1.27 (0.89, 1.81) | 0.186 |  |
| Sex |  |  |  |  |  | 0.270 |
| Male | Ref. | 0.86 (0.59, 1.26) | 0.90 (0.64, 1.27) | 1.26 (0.89, 1.80) | 0.289 |  |
| Female | Ref. | 1.03 (0.72, 1.42) | 1.28 (0.91, 1.80) | 1.34 (0.88, 2.04) | 0.089 |  |
| Hypertension |  |  |  |  |  | 0.027 |
| No | Ref. | 1.15 (0.76, 1.73) | 1.17 (0.78, 1.73) | 1.00 (0.68, 1.47) | 0.643 |  |
| Yes | Ref. | 0.75 (0.54, 1.05) | 1.04 (0.77, 1.42) | 1.47 (1.03, 2.09) * | 0.061 |  |
| Diabetes mellitus |  |  |  |  |  | 0.346 |
| No | Ref. | 1.03 (0.77, 1.39) | 1.11 (0.83, 1.43) | 1.30 (0.95, 1.77) | 0.159 |  |
| Yes | Ref. | 0.88 (0.54, 1.44) | 1.23 (0.77, 1.96) | 1.37 (0.80, 2.33) | 0.193 |  |
| Body mass index |  |  |  |  |  | 0.225 |
| < 30 | Ref. | 0.98 (0.74, 1.31) | 0.99 (0.72, 1.35) | 1.25 (0.91, 1.71) | 0.289 |  |
| ≥ 30 | Ref. | 0.87 (0.56, 1.36) | 1.30 (0.84, 2.02) | 1.58 (0.98, 2.56) | 0.047 |  |

Abbreviations: Q1, 0.10-0.50 g/dL; Q2, 0.51-0.60 g/dL; Q3, 0.61-0.80 g/dL; Q4, 0.81-2.22 g/dL; AAC, abdominal aortic calcification; OR, odd ratio; CI, confidence interval; Analysis was adjusted for age, sex, race/ethnicity, education level, marital status, family poverty income ratio, the complication of hypertension, diabetes mellitus, smoker, alcohol user, body mass index, waist circumference, the complication of coronary heart disease, congestive heart failure, angina pectoris, heart attack, and stroke, mean energy intake, calcium intake, and phosphorus intake, fast glucose, glycosylated hemoglobin, hemoglobin, total cholesterol, and triglyceride, high-density lipoprotein-cholesterol, blood urea nitrogen, serum uric acid, serum creatinine, estimated glomerular filtration rate, serum calcium, and phosphorus, lymphocyte, monocyte, mean cell volume, red cell distribution width, platelet, mean platelet volume, neutrophil-to-lymphocyte ratio, platelet-to-lymphocyte ratio, systemic immune inflammation index, system inflammation response index, albumin, gamma glutamyl transferase, alkaline phosphatase and serum iron.
