# Supplementary figures and images for: Association of Serum Total Bilirubin Level With Abdominal Aortic Calcification: A Population-Based Cross-Sectional Study
Source: Mediators Inflamm. 2025 Jul 27;2025:5229580. doi: 10.1155/mi/5229580 (PMC12318627; doi:10.1155/mi/5229580)

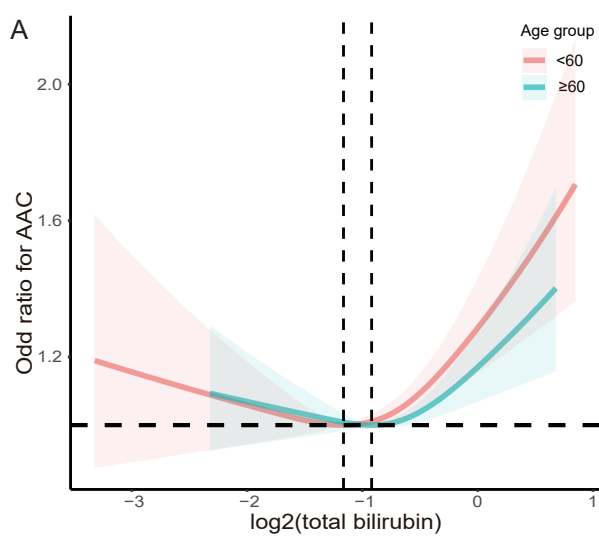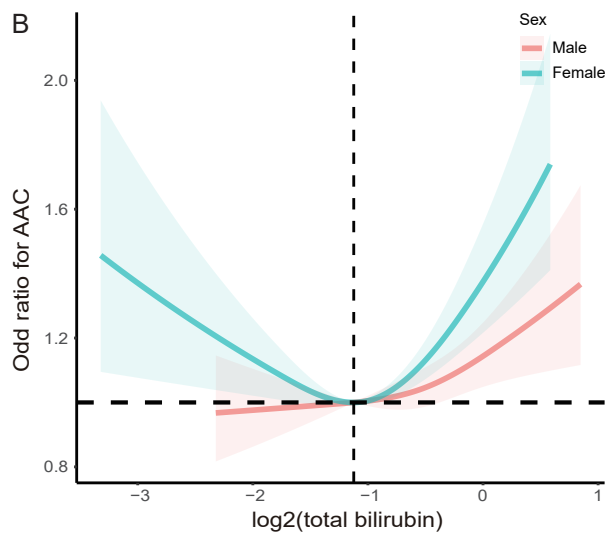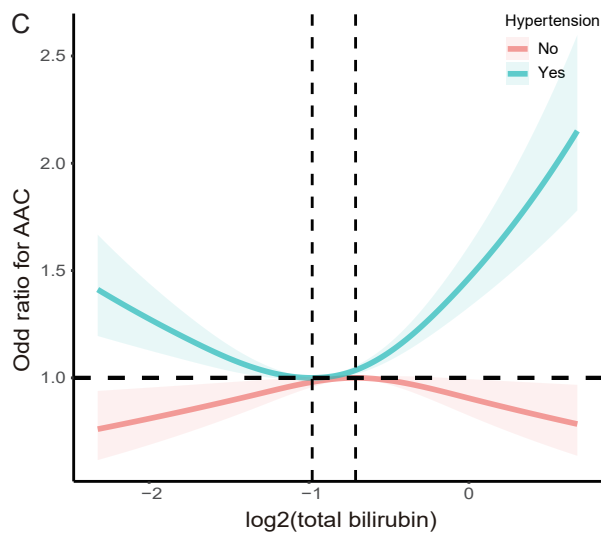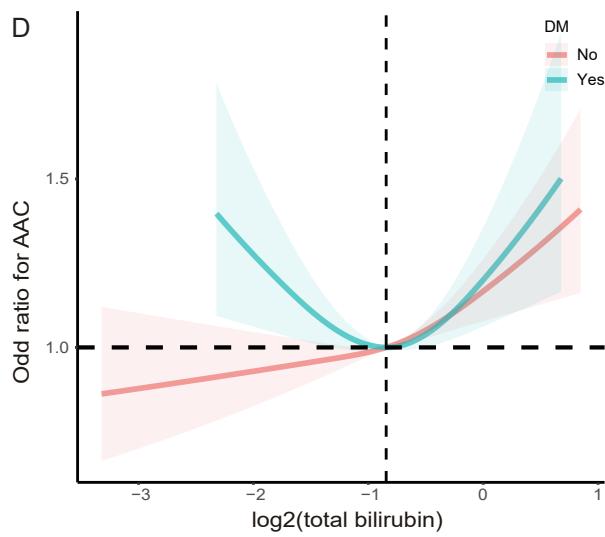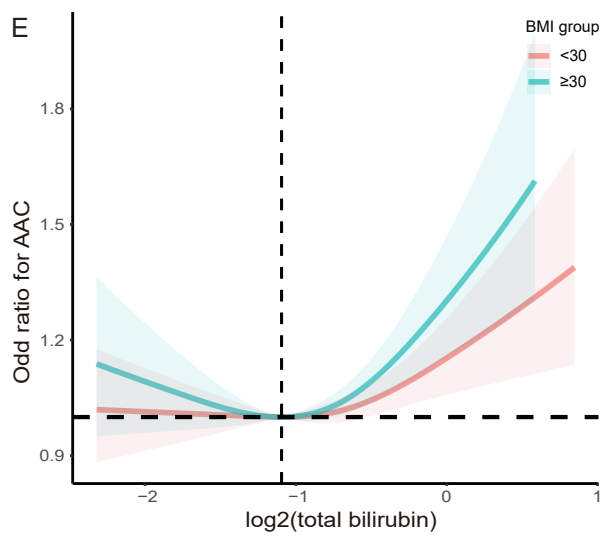

Supplement: Supporting Information 5 — Figure S1. Restricted cubic spline curve for the association of serum total bilirubin level with the risk of AAC stratified by (A) age, (B) sex, (C) hypertension, (D) DM, and (E) BMI. AAC, abdominal aortic calcification; BMI, body mass index; DM, diabetes mellitus. [file 5229580.f5.pdf]

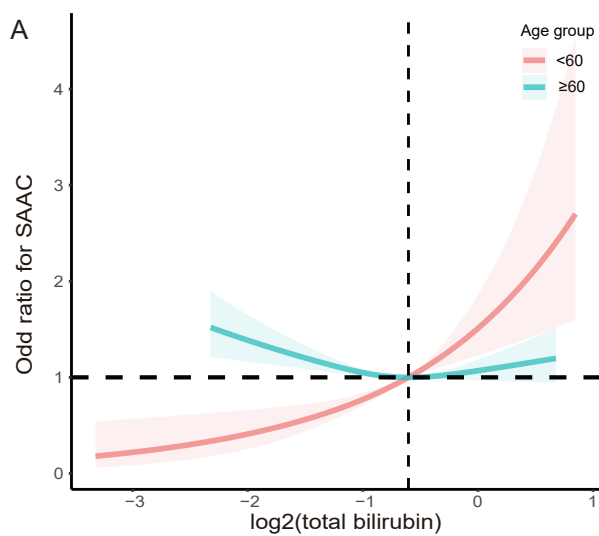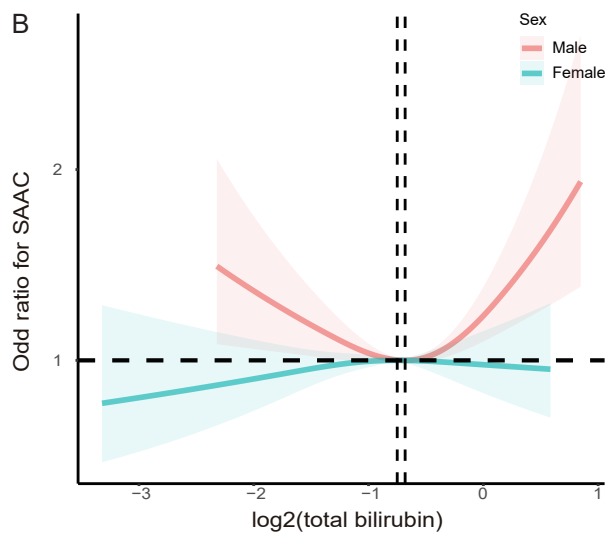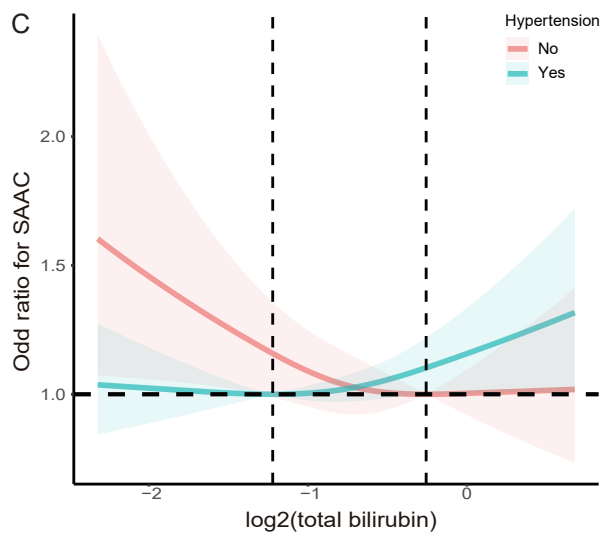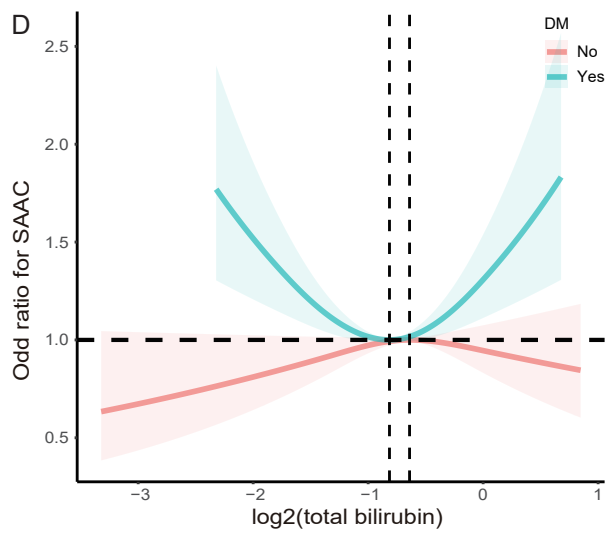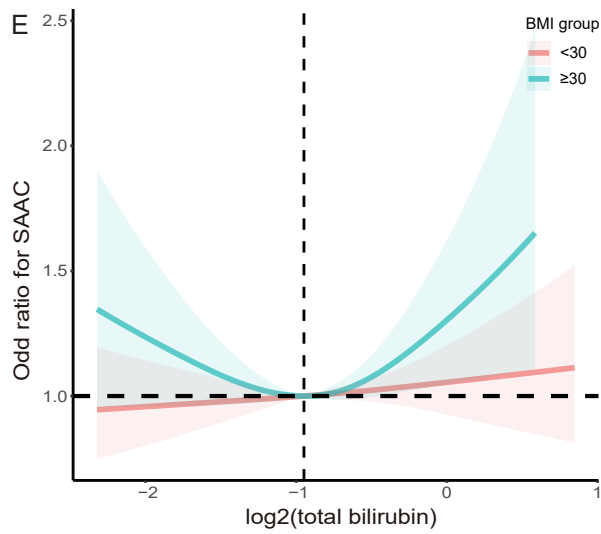

Supplement: Supporting Information 6 — Figure S2. Restricted cubic spline curve for the association of serum total bilirubin level with the risk of SAAC stratified by (A) age, (B) sex, (C) hypertension, (D) DM, and (E) BMI. BMI, body mass index; DM, diabetes mellitus; SAAC, severe abdominal aortic calcification. [file 5229580.f6.pdf]

A

IE=0.01173 ( $P=0.170$ )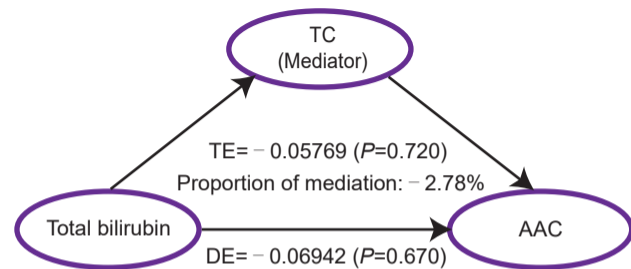

B

IE= - 0.0112 ( $P=0.130$ )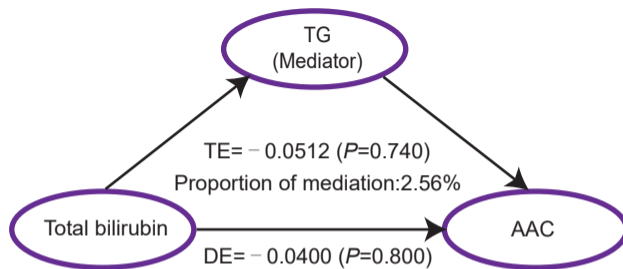

C

IE= - 0.0281 ( $P=0.048$ )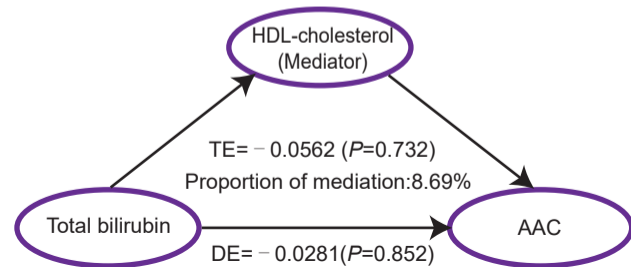

D

IE=0.01556 ( $P=0.220$ )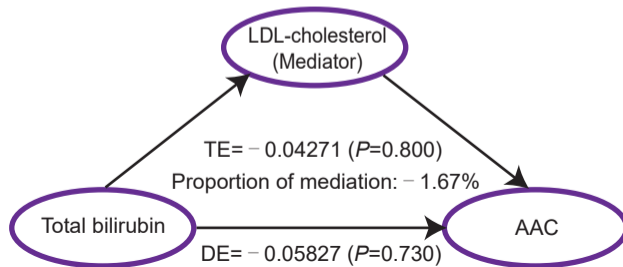

Supplement: Supporting Information 7 — Figure S3. Mediation analysis of lipid profiles on the interaction between total bilirubin and AAC. (A) mediation models of TC, total bilirubin, and AAC: Direct effect (TE = −0.05769; p=0.720) of total bilirubin (exposure) toward AAC (outcome), and TC mediation proportion is −2.78%; indirect effect (IE = 0.01173; p=0.170) of total bilirubin (exposure) toward TC (mediator) and effect AAC (DE = −0.06942; p=0.670), from TC (mediator) toward AAC (outcome). (B) Mediation models of TG, total bilirubin, and AAC: direct effect (TE = −0.0512; p=0.740) of total bilirubin (exposure) toward AAC (outcome), and TG mediation proportion is 2.56%; indirect effect (IE = −0.0112; p=0.130) of total bilirubin (exposure) toward TG (mediator) and effect AAC (DE = −0.0400; p=0.800), from TG (mediator) toward AAC (outcome). (C) Mediation models of HDL-cholesterol, total bilirubin, and AAC: Direct effect (TE = −0.0562; p=0.732) of total bilirubin (exposure) toward AAC (outcome), and HDL-cholesterol mediation proportion is 8.69%; indirect effect (IE = −0.0281; p=0.048) of total bilirubin (exposure) toward HDL-cholesterol (mediator) and effect AAC (DE = −0.0281; p=0.852), from HDL-cholesterol (mediator) toward AAC (outcome). (D) Mediation models of LDL-cholesterol, total bilirubin, and AAC: direct effect (TE = −0.04271; p=0.800) of total bilirubin (exposure) toward AAC (outcome), and LDL-cholesterol mediation proportion is −1.67%; indirect effect (IE = 0.01556; p=0.220) of total bilirubin (exposure) toward LDL-cholesterol (mediator) and effect AAC (DE = −0.05827; p=0.730), from LDL-cholesterol (mediator) toward AAC (outcome). AAC, abdominal aortic calcification; high density lipoprotein-cholesterol; LDL-cholesterol, low density lipoprotein-cholesterol; TC, total cholesterol; TG, triglycerides. [file 5229580.f7.pdf]

A

$$IE = 0.001136 \ (P = 0.170)$$

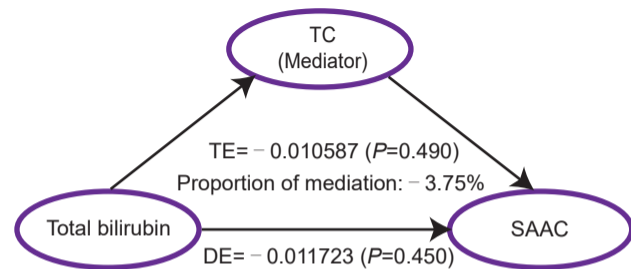

B

$$IE = -0.000742 \ (P = 0.180)$$

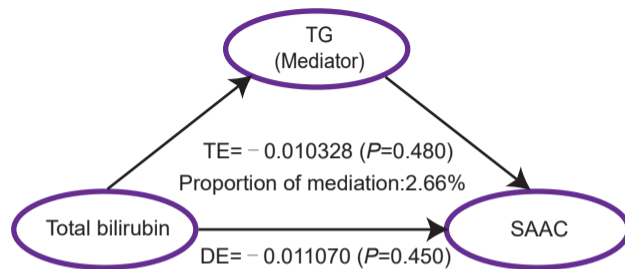

C

$$IE = -0.00217 \ (P = 0.058)$$

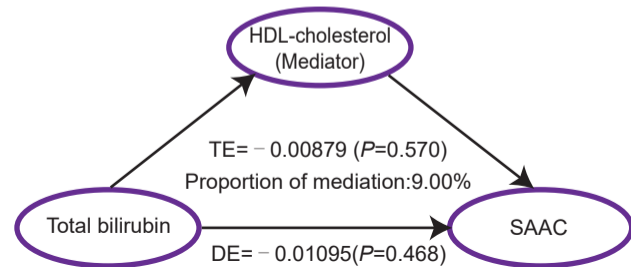

D

$$IE = 0.001404 \ (P = 0.160)$$

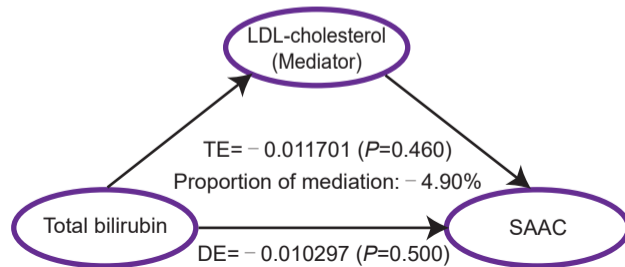

Supplement: Supporting Information 8 — Figure S4. Mediation analysis of lipid profiles on the interaction between total bilirubin and SAAC. (A) Mediation models of TC, total bilirubin, and SAAC: Direct effect (TE = −0.010587; p=0.490) of total bilirubin (exposure) toward SAAC (outcome), and TC mediation proportion is −3.75%; indirect effect (IE = 0.001136; p=0.170) of total bilirubin (exposure) toward TC (mediator) and effect SAAC (DE = −0.011723; p=0.450), from TC (mediator) toward SAAC (outcome). (B) Mediation models of TG, total bilirubin, and SAAC: Direct effect (TE = −0.010328; p=0.480) of total bilirubin (exposure) toward SAAC (outcome), and TG mediation proportion is 2.66%; indirect effect (IE = −0.000742; p=0.180) of total bilirubin (exposure) toward TG (mediator) and effect SAAC (DE = −0.011070; p=0.450), from TG (mediator) toward SAAC (outcome). (C) Mediation models of HDL-cholesterol, total bilirubin, and SAAC: Direct effect (TE = −0.00879; p=0.570) of total bilirubin (exposure) toward SAAC (outcome), and HDL-cholesterol mediation proportion is 9.00%; indirect effect (IE = −0.00217; p=0.058) of total bilirubin (exposure) toward HDL-cholesterol (mediator) and effect SAAC (DE = −0.01095; p=0.468), from HDL-cholesterol (mediator) toward SAAC (outcome). (D) Mediation models of LDL-cholesterol, total bilirubin, and SAAC: Direct effect (TE = −0.011701; p=0.460) of total bilirubin (exposure) toward SAAC (outcome), and LDL-cholesterol mediation proportion is −4.90%; indirect effect (IE = 0.001404; p=0.160) of total bilirubin (exposure) toward LDL-cholesterol (mediator) and effect SAAC (DE = −0.010297; p=0.500), from LDL-cholesterol (mediator) toward SAAC (outcome). HDL-cholesterol, high density lipoprotein-cholesterol; LDL-cholesterol, low density lipoprotein-cholesterol; SAAC, severe abdominal aortic calcification; TC, total cholesterol; TG, triglycerides. [file 5229580.f8.pdf]
